# Towards a comprehensive characterization of spatio-temporal dependence of light-induced electromagnetic forces in dielectric liquids

**DOI:** 10.1038/s41598-024-56176-1

**Published:** 2024-03-07

**Authors:** N. G. C. Astrath, E. V. Bergmann, B. Anghinoni, G. A. S. Flizikowski, A. Novatski, C. Jacinto, T. Požar, M. Kalin, L. C. Malacarne, M. L. Baesso

**Affiliations:** 1 Department of Physics, Universidade Estadual de Maringá, Maringá, PR 87020-900 Brazil; 2 School of Electrical Engineering and Computer Science, University of Ottawa, Ottawa, ON K1N6N5 Canada; 3 Department of Physics, Universidade Estadual de Ponta Grossa, Ponta Grossa, PR 84030-900 Brazil; 4 Institute of Physics, Universidade Federal de Alagoas, Maceió, AL 57072-900 Brazil; 5 Faculty of Mechanical Engineering, University of Ljubljana, 1000 Ljubljana, Slovenia

**Keywords:** Optical physics, Applied physics, Acoustics

## Abstract

The interaction of localized light with matter generates optical electrostriction within dielectric fluids, leading to a discernible change in the refractive index of the medium according to the excitation’s light profile. This optical force holds critical significance in optical manipulation and plays a fundamental role in numerous photonic applications. In this study, we demonstrate the applicability of the pump-probe, photo-induced lensing (PIL) method to investigate optical electrostriction in various dielectric liquids. Notably, the thermal and nonlinear effects are observed to be temporally decoupled from the electrostriction effects, facilitating isolated observation of the latter. Our findings provide a comprehensive explanation of optical forces in the context of the recently introduced microscopic Ampère electromagnetic formalism, which is grounded in the dipolar approximation of electromagnetic sources within matter and characterizes electrostriction as an electromagnetic-induced stress within the medium. Here, the optical force density is re-obtained through a new Lagrangian approach.

## Introduction

It is long-known from classical electromagnetism that electromagnetic fields can store linear momentum. Likewise, it is also known that the effects of electromagnetic fields can only be perceived through their coupling with matter, where momentum and energy transfers between the two systems take place. Such primordial transfer phenomena are, however, not completely understood to this date—a fact directly related to the century-old Abraham-Minkowski dilemma on the photonic momentum inside materials^1–6^. Although consistent development has been achieved by elucidating the difference between kinetic and canonical momenta in continuum field+matter systems^7,8^, the electromagnetic force density intrinsically associated with the closed momentum continuity equation also possesses stress terms, which, on their turn, have not been unambiguously settled to date. Therefore, distinct electromagnetic force densities exist in the literature^9^, and distinguishing among them on experimental grounds has historically been challenging, especially at optical frequencies^10^.

Among the numerous possibilities of light-matter interactions that can take place when laser beams illuminate a sample, there exists a force compressing the material towards the regions of higher beam intensity. This effect is known as electrostriction for polarizable matter and magnetostriction for magnetizable matter and occurs in both fluids and solids, regardless of their underlying microscopic structure. Being a consequence of the electromagnetic forces acting in the bulk medium, these striction effects share the general problem described in the last paragraph, i.e., they are still not theoretically well understood. Moreover, the observation of striction effects is often challenging because they are overshadowed by the predominance of absorption processes, which typically exhibit greater magnitude^11^. Controlling this interplay between opto-mechanical and thermal effects is of great interest to many modern technologies—especially for applications in optical trapping^12–16^, photonics^17^, optofluidics^18,19^ and biological systems^20–23^. In fact, it is long-known that striction forces play no role in the dynamics of the center-of-mass of illuminated samples^5^, being then frequently neglected in optical-based manipulations of rigid bodies. Nevertheless, they are essential in local force and energy considerations, generating significant deformation effects on elastic matter samples like biological cells^24^.

One suitable choice to probe the optical electrostriction effect is to adopt transparent non-magnetic dielectric fluids as the sample medium. This allows the observation of, for example, the small changes in the refractive index of the medium due to the compressing forces from electrostriction (providing thermal effects can be suppressed). Indeed, the spatio-temporal behavior of the optical electrostriction generated in bulk material has been recently reported in two works using water^25,26^, where the non-linear and thermal effects could be carefully decoupled in time in the photo-induced lensing (PIL) experimental technique employed. Additionally, the results have shown an excellent agreement with the optical force density from the so-called microscopic Ampère formulation, where the electrostriction effect is interpreted as a force density contribution stemming from the bulk stress generated by the interaction of the external fields with the induced electric dipoles in the material^27,28^.

To confirm that the microscopic Ampère form of the coupling between light and matter matches the observation obtained with water, we experimentally expand the hosting medium to additional six different liquids. The optical electrostriction effect in transparent dielectric liquids is measured using a photo-induced lensing experimental technique. The results are all very well modeled by the microscopic Ampère force density, reinforcing the interpretation of the electrostriction effect as an electromagnetically-induced bulk stress and providing further evidence for the validity of the proposed theoretical model.

## Results

### Optical force in the dielectric liquids

The dielectric medium is described as a continuum of point molecules, initially at rest, with electric dipole moment $\textbf{p}$ and magnetic dipole moment $\textbf{m}$, which can both be induced by external optical fields. When such fields are applied, the Lagrangian ${\mathcal {L}}$ of each molecule is given in the laboratory inertial reference frame as

1 $$\begin{aligned} {\mathcal {L}} = \frac{1}{2}m|\textbf{v}|^2 +\left[ \textbf{p} (\textbf{E})+ \frac{1}{c^2}\textbf{v}\times \textbf{m}(\textbf{B})\right] \cdot \textbf{E} +\left[ \textbf{m} (\textbf{B}) - \textbf{v}\times \textbf{p}(\textbf{E})\right] \cdot \textbf{B}, \end{aligned}$$

where *m* is the mass of an individual molecule, *c* is the speed of light in vacuum, $\textbf{E}$ is the electric field, $\textbf{B}$ is the magnetic induction field, and $\textbf{v}$ is the non-relativistic molecule’s velocity acquired due to the interaction with the external fields. Lagrangians similar to Eq. (1) have appeared in earlier literature, for example, for static fields^29^ and induced dipoles at rest^30^. Here, we consider the induced dipoles to be linear in the corresponding external fields, i.e., we take $\textbf{p} \propto \textbf{E}$ and $\textbf{m} \propto \textbf{B}$, leading to the optical force $\textbf{F}$ acting on each molecule described in the laboratory frame (see Methods)

2 $$\begin{aligned} \textbf{F}=\frac{1}{2} \varvec{\nabla }(\textbf{p} \cdot \textbf{E} + \textbf{m} \cdot \textbf{B}) +\frac{\,\textrm{d}}{\,\textrm{d}t}\left( \textbf{p}\times \textbf{B}-\frac{1}{c^2}\textbf{m}\times \textbf{E}\right) , \end{aligned}$$

which yields in the continuum medium description the so-called microscopic Ampère optical force density^25,28^, namely

3 $$\begin{aligned} \textbf{f}= -\frac{1}{2}|\textbf{E}|^2\varvec{\nabla }\varepsilon -\frac{1}{2}|\textbf{H}|^2\varvec{\nabla }\mu +\frac{1}{2}\varvec{\nabla }\left(\textbf{P} \cdot \textbf{E} + \textbf{M} \cdot \textbf{B}\right)\ +\frac{n^2-1}{c^2}\frac{\partial }{\partial t}(\textbf{E} \times \textbf{H}). \end{aligned}$$

Here, $\varepsilon$ and $\mu$ are the medium’s permittivity and permeability, respectively, *n* is the medium’s refractive index, *t* is time, $\textbf{H}$ is the magnetic field, $\textbf{P}$ is the polarization field and $\textbf{M}$ is the magnetization field.

**Figure 1:**
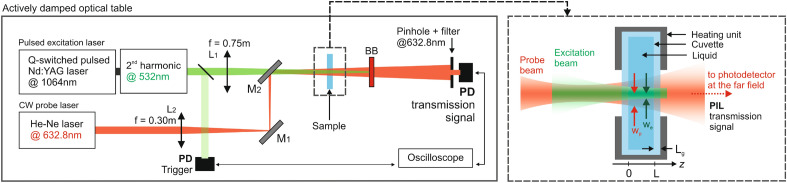
Schematic drawing of the experimental photo-induced lensing (PIL) setup. A pulsed excitation laser beam is focused onto the sample. A continuous-wave laser is aligned along the same axis as the excitation beam to capture the focusing/defocusing effects. The change in intensity at the center of the probe beam, following transmission through the sample, is detected using a pinhole-laser line filter-photodetector assembly in the far-field. A digital oscilloscope, triggered by the photodiode (PD), recorded data at a 10 Hz repetition rate for the pulsed excitation. The sample’s temperature is maintained at $298.15 \pm 0.05$ K. Details are given in Methods.

Equation (3) is valid for linear, non-dispersive and lossless dielectrics. The first and second terms act in non-homogeneous regions and are associated with the surface deformations in homogeneous samples, as shown for example in Refs. ^25,31–34^. The third and fourth terms are the electro- and magnetostriction effects, having the force density larger in the regions of higher beam intensity. At last, the time-derivative term in Eq. (3) is known as the Abraham force (density) and is directly related to the optical linear momentum transfer inside the dielectric^4,5,9^.

### Equations governing temperature and pressure fields

Under pulsed excitation, the optical electrostriction force acts as a compressing force in the dielectric liquid generating a local pressure distribution. Residual heat deposition also contributes to this change. The samples investigated here are low-absorbing dielectric liquids. This allows us to consider the laser beam intensity and profile to be constant along the sample thickness (*z*–axis). With no dependence on *z*, the problem acquires a cylindrical symmetry and the intensity of the applied Gaussian beam can then be written as a function of only the radial coordinate *r* and time *t* as

4 $$\begin{aligned} I(r,t) = Q_0 {\textrm{e}}^{-\frac{2r^2}{w_{{\textrm{e}}} ^2}}h(t), \end{aligned}$$

where $Q_0 = 2 E_{\textrm{ p}}/\pi w_{{\textrm{e}}} ^2$ is the heat source, with $E_{\textrm{ p}}$ and $w_{{\textrm{e}}}$ being the excitation beam energy and waist, respectively. The function *h*(*t*) describes the time-dependence of the laser pulse and is given as $h(t)=t_0^{-1}\textrm{exp}[-(t-\xi )^2/\tau ^2]$, where the time of maximum irradiance and width are denoted by $\xi$ and $\tau$, respectively, and $t_0 = \tau \sqrt{\pi }[1+\textrm{Erf}(\xi /\tau )]/2$ is the normalization parameter given in terms of the error function $\textrm{Erf}(x)$.

On their turn, the bulk temperature field *T*, pressure field *p* and optical force density $\textbf{f}$ are also only dependent on *r* and *t*. Assuming negligible viscosity, *T*(*r*, *t*) and *p*(*r*, *t*) can be obtained from a set of coupled differential equations governing the temperature diffusion and the pressure relaxation in terms of elastic waves, namely^35^

5 $$\begin{aligned} \rho _\mathrm m c_p\frac{\partial T(r,t)}{\partial t}-k\varvec{\nabla }^2 T(r,t)=A_{{\textrm{e}}} I(r,t) \end{aligned}$$

and

6 $$\begin{aligned} \varvec{\nabla }^2 p(r,t) -\frac{1}{c_{\textrm{ s}} ^2}\frac{\partial ^2 p(r,t)}{\partial t^2}=\varvec{\nabla }\cdot \textbf{f}(r,t) -\frac{\beta A_{{\textrm{e}}}}{c_p} \frac{\partial I(r,t)}{\partial t}, \end{aligned}$$

where $\rho _\mathrm m$ is the mass density, $c_p$ is the specific heat at constant pressure, *k* is the thermal conductivity, $c_{\textrm{ s}}$ is the sound velocity, $\beta$ is the volumetric thermal expansion coefficient, and $A_{{\textrm{e}}}$ is the absorption coefficient at the excitation’s beam wavelength.

For bulk, non-magnetic fluids under optical excitation, only the third term in Eq. (3) contributes significantly to the optical force. In terms of beam intensity, we have

7 $$\begin{aligned} \textbf{f}(r,t) = \frac{2}{c}\frac{n-1}{n+1} \varvec{\nabla }_r I(r,t), \end{aligned}$$

where $\varvec{\nabla }_r$ denotes the gradient operator in the radial direction. Equations (5) and (6) have semi-analytical solutions, given in Methods.

### Photo-induced lensing method

A schematic illustration of the experiment is presented in Fig. 1, while the details of the setup are described in Methods. In the measurements, the samples are irradiated with a nanosecond laser pulse causing changes in local pressure due to the optical forces and a small contribution from heat deposition. At this time scale, the nonlinear optical Kerr effect also contributes to the generated signal. A low-intensity laser is used to probe the induced effects by monitoring the wavefront in transmission distortion in the far-field by a fast photodetector.

The temperature and pressure variations change the local characteristics of the fluids according to their spatio-temporal behavior. Additionally, the high beam intensity of the pulsed excitation also contributes to nonlinear processes. In this work, we are specifically interested in the small changes of the local refractive index in the bulk medium, which are in our model generated via four distinct physical mechanisms: (i) the temperature rise due to optical absorption, the pressure variation due to (ii) the optical force and (iii) thermally-induced stresses, and (iv) the nonlinear interaction with the dipoles due to the high beam intensity distribution. These contributions are described, accordingly, in terms of (i) the thermo-optic coefficient $\partial n/\partial T$, (ii-iii) the piezo-optic coefficient $\partial n/\partial p$, and (iv) the effective Kerr effect nonlinear refractive index, $n_{2,\textrm{eff}} = \partial n/\partial I$. Assuming small perturbation of the refractive index and using a general expansion to first-order, the local change in the medium’s refractive index is then

8 $$\begin{aligned} \Delta n (r,t) = \frac{\partial n}{\partial T} T(r,t)+\frac{\partial n}{\partial p} p(r,t)+\frac{\partial n}{\partial I} I(r,t). \end{aligned}$$

The changes of the refractive index with temperature $\partial n/\partial T$, pressure $\partial n/\partial p$ and light intensity $\partial n/\partial I$ are evaluated at the probe wavelength, while the temperature field *T*(*r*, *t*), pressure field *p*(*r*, *t*) and light intensity field *I*(*r*, *t*) arise due to the interaction of the excitation laser with matter. Specifically, the optical force density corresponds to a source term in the acoustic wave equation shown in Eq. (6)—therefore, it is directly related to the pressure field *p*(*r*, *t*) in Eq. (8). The variation of the local refractive index $\Delta n$ will cause a probe beam to acquire a spatio-temporal dependent phase shift as it propagates through the sample. As $\Delta n$ does not depend on *z*, this phase shift is given by $\Phi (r,t) = (2 \pi /\lambda _{\textrm{ p}} )L\Delta n(r,t)$, where $\lambda _{\textrm{ p}}$ is the probe beam wavelength inside the sample and *L* is the sample thickness. The intensity of the probe beam will, in turn, vary in time due to $\Phi (r,t)$. It can be shown that using Fresnel diffraction theory, the PIL signal *S*(*t*) is proportional to the intensity of the center of the probe beam measured at the detector plane placed in the far-field region and is given by^36^

9 $$\begin{aligned} S(t) = \left| \int _0^{\infty }\frac{2 r}{w_{\textrm{ p}} ^2} \textrm{exp}\left[ -(1+\mathrm i V)\frac{r^2}{w_{\textrm{ p}} ^2}-\mathrm i \Phi (r,t)\right] \!\!\,\textrm{d}r\right| ^2, \end{aligned}$$

where *V* is an experimental parameter and $w_{\textrm{ p}}$ is the probe beam’s waist in the sample. To obtain *S*(*t*), the semi-analytical expressions for *T*(*r*, *t*) and *p*(*r*, *t*) are employed (see Methods).

**Figure 2:**
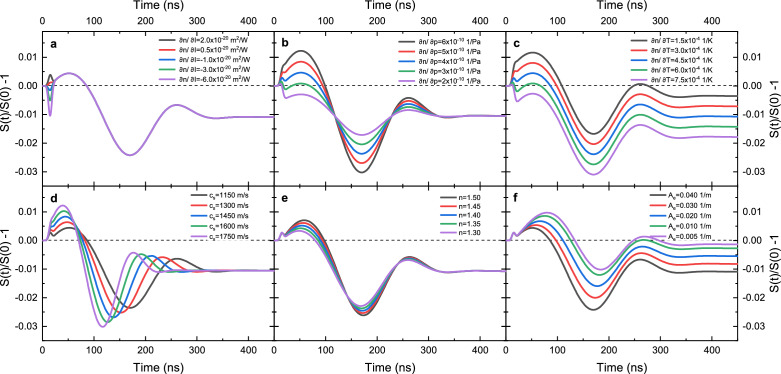
Sensitivity study of the normalized PIL signal by changing physical properties of the sample. The transient signals were calculated using the physical properties of ethanol (see Supplementary Table S1) and changing (**a**) the nonlinear refractive index (affecting only $S_{\textrm{Kerr}}$), (**b**) the piezo-optic coefficient (affecting both $S_{\textrm{ESW}}$ and $S_{\textrm{TEW}}$), (**c**) the thermo-optic coefficient (affecting only $S_{\textrm{TD}}$), (**d**) the speed of sound (affecting both $S_{\textrm{ESW}}$ and $S_{\textrm{TEW}}$), (**e**) the refractive index (affecting only $S_{\textrm{ESW}}$), and (**f**) the optical absorption coefficient (affecting both $S_{\textrm{TEW}}$ and $S_{\textrm{TD}}$). Note that changing the specific heat has the same effect as changing the reciprocal value of the optical absorption coefficient. Additionally, the volumetric thermal expansion coefficient only affects $S_{\textrm{TEW}}$, while the mass density directly affects $S_{\textrm{TD}}$, but also $S_{\textrm{ESW}}$ and $S_{\textrm{TEW}}$ because the speed of sound depends on it.

The transient PIL signal shows the convoluted evolution of the probe signal. The probe beam detects the entire area influenced by the excitation laser, and the complex transmitted pattern of the probe beam just out of the sample propagates to the detector plane. The intensity fluctuation detected at the probe beam’s central point in the far-field results from combining all the refractive index influencing effects within the liquid. The distortion of the probe beam’s wavefront arises from the non-uniform interaction of the excitation beam with the sample, leading to an increase in internal energy. This increased internal energy is then dissipated in two distinct modes of hydrodynamic relaxation. Consequently, the rise in internal energy induces a temperature change within the sample or any adjacent coupling material. If it occurs more rapidly than the time required for the fluid to either expand or contract, this temperature alteration leads to a rapid change in pressure. The pressure perturbation subsequently dissipates by generating an acoustic wave, known as thermoelastic wave (TEW). Once the pressure has returned to equilibrium, density and refractive index changes proportional to the temperature persist.

### Theoretical sensitivity study of the PIL signal

Interpretation of the PIL signal (Eq. (9)) can be significantly simplified if it is first recast in a normalized way as $(S(t)/S(0)-1)$ and then linearized assuming small-perturbation approximation

10 $$\begin{aligned} \left( \frac{S(t)}{S(0)}-1 \right) \approx \left( \frac{S_{\textrm{TD}}(t)}{S(0)}-1 \right) + \left( \frac{S_{\textrm{ESW}}(t)}{S(0)}-1 \right) + \left( \frac{S_{\textrm{TEW}}(t)}{S(0)}-1 \right) + \left( \frac{S_{\textrm{Kerr}}(t)}{S(0)}-1 \right) , \end{aligned}$$

where *S*(0) is the initial, steady PIL signal before the excitation, $S_{\textrm{TD}}(t)$ is the PIL signal solely caused by the thermal deposition, $S_{\textrm{ESW}}(t)$ is the piezo-optic PIL signal uniquely caused by the propagation of the electrostriction-generated elastic waves (ESW), $S_{\textrm{TEW}}(t)$ is the piezo-optic PIL signal reflecting only the launch of the thermally-generated elastic waves (TEW), and lastly $S_{\textrm{Kerr}}(t)$ is the short-lived PIL signal exclusively due to Kerr effect. See Supplementary Information for the definitions of individual contributions which appear in Eq. (10).

Finally, each constituent is separately analyzed. The Kerr contribution $(S_{\textrm{Kerr}}(t)/S(0)-1)$ is non-zero only at the very beginning when the medium is being illuminated by the excitation beam. The electrostrictive relaxation $(S_{\textrm{ESW}}(t)/S(0)-1)$ is observable during the time needed for the electrostriction-launched elastic waves to exit the probing volume and stems exclusively from the third term in Eq. (3), which is for our system simplified as Eq. (7) and used as the source term in Eq. (6). Thermal relaxation $(S_{\textrm{TEW}}(t)/S(0)-1)$ also occurs as the emission of an elastic wave, while the signal due to thermal deposition $(S_{\textrm{TD}}(t)/S(0)-1)$ builds up during the sample illumination time and then persists, as thermal diffusion acts on much longer time scales.

Apart from the parameters pertaining to the excitation and detection laser beams, and the geometry of the setup, each of the PIL signal constituents depends on different properties of the liquid. The Kerr contribution is a function of $(\partial n/\partial I)$ and has a shape closely resembling the temporal shape of the excitation beam intensity *I*(*t*). The relaxation of the generated stresses due to electrostriction is a function of $(\partial n/\partial p, n, c_{\textrm{s}})$, having a more involved peak-trough-peak signature. The relaxation of the thermally-induced stresses is a function of $(\partial n/\partial p, \beta A_{\textrm{e}} / c_p, c_{\textrm{s}})$, having a shape equal to the time derivative of the electrostriction-generated waves^35^. Neglecting the initial rapid energy deposition on the excitation beam path which depends on the actual temporal distribution of the laser pulse, the thermal signal is a function of $(\partial n/\partial T, A_{\textrm{e}}/(\rho _{\textrm{m}} c_p))$ and can be observed as a steady negative offset.

The thermo-optical and mechanical properties of the sample thus dictate the shape of the time-dependent PIL signal. Figure 2 illustrates how the properties exposed in the previous paragraph affect the transient signals. They were simulated using Eq. (9), as a normalized PIL signal $(S(t)/S(0)-1)$, starting with the physical properties of ethanol, as given in Supplementary Table S1 and Ref. ^37^, but separately changing each of the influencing properties.

**Figure 3:**
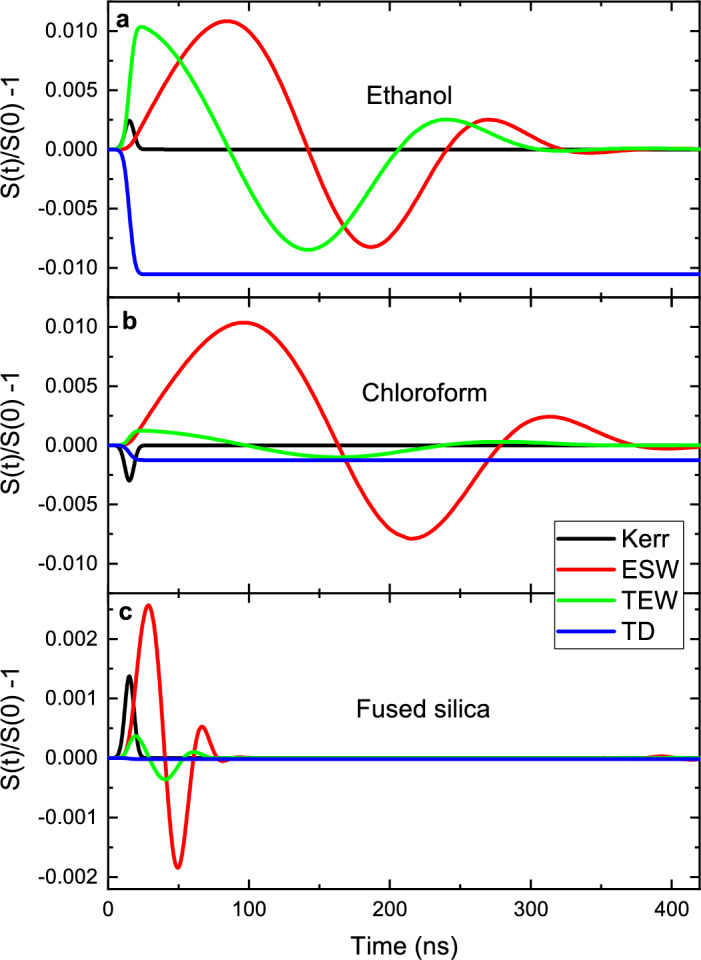
Decomposition of the normalized PIL signal. Numerical calculations of the individual components in (**a**) ethanol, (**b**) chloroform, and (**c**) quartz cuvette walls showing the signal due to radiation forces along with contributions of the thermal deposition and optical Kerr effects.

Figure 2a shows the effect of the variation of the nonlinear refractive index. Positive values of the nonlinear refractive index form convergent lensing and thus positive signal in the first 20 ns of the transient. On the other hand, negative values generate a divergent field of the refractive index, producing a negative initial pulse in the normalized PIL signal.

Figure 2b depicts the influence of the piezo-optic coefficient on the normalized PIL signal. The amplitude of both types of elastic waves (ESW and TEW) linearly scales with the change of the piezo-optic coefficient.

The thermo-optic coefficient directly affects the normalized PIL signal’s negative offset. This is clearly seen in Fig. 2c.

The speed of sound has an obvious effect on the propagation velocity of both types of elastic waves. The faster-moving waves exit the probing volume at shorter times and also give rise to larger amplitudes of their peak-trough-peak signature in the normalized PIL signal, which is a clear observation of Fig. 2d.

As the refractive index only affects electrostriction, it is thus primarily detected in the amplitude of the electrostriction-generated elastic wave. The reason why the peak-trough-peak signature shifts to larger times is due to the constant, but non-negligible contribution of the thermoelastic wave. This is conveyed in Fig. 2e.

Lastly, Fig. 2f displays the variation of the optical absorption coefficient which affects both, the amplitude of the thermoelastically-driven elastic relaxation as well as the amount of the heat deposition of the excitation light which corresponds to the amplitude of the negative signal offset. Increasing the optical absorption coefficient shifts the entire transient to more negative values.

The contribution from each effect to the PIL signal can be calculated separately as shown in Fig. 3. The numerical calculations of the individual optical forces are presented for ethanol, chloroform, and the cuvette walls. Note that the amplitudes of the transients are much larger for the liquid, dominating the total time-dependent signal observed. The Kerr effect, $(S_{\textrm{Kerr}}/S(0)-1)$, appears only during the pulse duration. The electrostriction, $(S_{\textrm{ESW}}/S(0)-1)$, and thermal, $(S_{\textrm{TEW}}/S(0)-1)$, waves oscillate out of phase, governing the total PIL signal. The thermal deposition effect, $(S_{\textrm{TD}}/S(0)-1)$, shifts the total signal to negative values. Note that $(S_{\textrm{ESW}}/S(0)-1)$ is dominant over the thermal effects for chloroform, which has a very low optical absorption coefficient.

The interpretation tool provided by this sensitivity analysis allows one to better understand and decode the experimental PIL signals obtained with actual low-loss liquids.

### Experimental PIL signal

**Figure 4:**
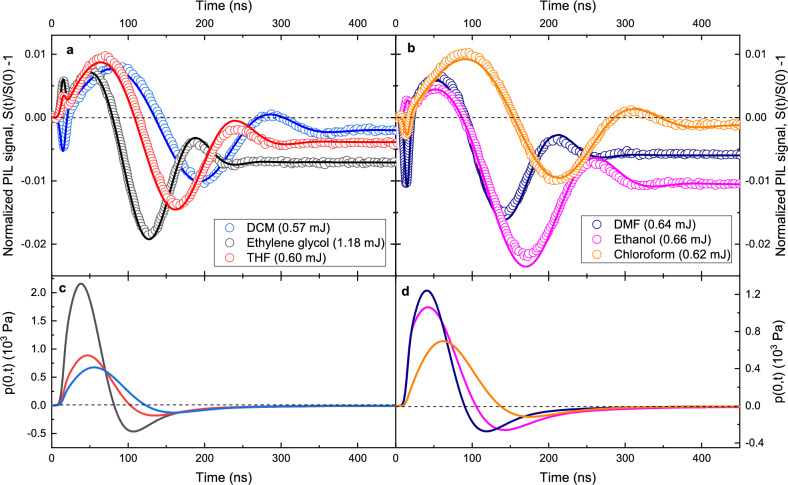
Time-dependent PIL transients. (**a**, **b**) Normalized PIL signal under pulsed laser excitation at 532 nm. The transients show the intensity variation of the center of a continuous probe laser beam transmitted through the cuvette-liquid interfaces measured by a photodetector in the far-field. Open symbols are experimental data and continuous lines represent the numerical calculations using *S*(*t*); confidence level of 95%. The uncertainties in (**a**) and (**b**) are smaller than 1% and correspond to the standard deviation of the mean over all the experiments (see Methods). The optical path length of the cuvette was $L = 5$ mm with the cuvette walls having a thickness of $L_\mathrm g = 1.25$ mm. (**c**, **d**) Total pressure calculated at the center of the liquid samples with contributions due to optical forces and thermally-induced stresses.

We have performed experiments using six low-loss dielectric liquids: ethanol, chloroform, ethylene glycol, dimethylformamide (DMF), dichloromethane (DCM), and tetrahydrofuran (THF), whose properties are collected in Supplementary Table S1. The experimental time-dependent photo-induced lensing signals of these liquids including the contribution of the fused silica walls of the cuvette at both ends are presented in Fig. 4a,b (open symbols).

Calculating the photo-induced lensing signal requires determining the temperature and pressure fields while taking into account all the effects of optical forces within both the liquid and the cuvette walls (continuous lines in Fig. 4). The semi-analytical predictions are in excellent agreement with the experimental transients. It shows quantitatively that the effects of optical forces in liquids can be fully described by Eqs. (4), (20) and (21). The only fitting parameters for the liquids are the effective nonlinear refractive index, $n_{2,\textrm{eff}}$, and the optical absorption coefficient at 532 nm. These parameters are also given in Supplementary Table S1.

Figure 4c,d show the calculated pressure changes over time at the center of the liquid column using *p*(0, *t*). They illustrate the combined shape and amplitude of the kPa-large pressure waves due to the relaxation of thermal and electrostriction-induced stresses. Notice that we used twice as large excitation pulse energy for the interaction with the ethylene glycol compared to the other five liquids to produce nearly the same amplitude of the peak-trough-peak part of the signal, corresponding to the relaxation in the form of elastic waves. The main reason for this is a low value of $\partial n / \partial p$ for ethylene glycol compared to the other five liquids.

Both, electrostriction forces and the tendency of the liquid to expand when heated give rise to a positive pressure build-up. The pressure transient thus commences its propagation as a purely monopolar compression wave. This perturbation relaxes cylindrically outwards and towards the symmetry axis as two compression waves, one converging and the other diverging away from the symmetry axis. The evolution of pressure on the symmetry axis *p*(0, *t*) is affected solely by the converging pressure wave. Its maximum occurs at the time when the maximum of the initial pressure build-up in the first 20 ns reaches the symmetry axis. After the wave passes this axis, it transforms from a half-cycle, monopolar compression pulse to a full single-cycle, compression-tension wave. This phenomenon is indicative of the Gouy phase shift effect which was also observed by other authors in the case of focusing of surface and bulk ultrasonic waves in various configurations^38,39^. In cylindrical geometry, the effect of Gouy $\pi /2$ phase shift when applied to a half-cycle (monopolar) compression pulse is to broaden the pulse by approximately a factor of 2 and transform the shape into a single-cycle (bipolar) one, as in Ref. ^39^. This is why the evolution of the axial pressure, presented in Fig. 4c,d, features an initial large-amplitude compression pulse followed by a small-amplitude tension tail.

## Discussion

At the nanosecond time scale, the refractive index of the samples presents a strong dependence on the optical intensity. This intensity-dependent refractive index or nonlinear index of refraction, $n_2$, gives the rate at which the index changes with the laser intensity, known as the optical Kerr effect. The measured normalized Kerr signal $(S_{\textrm{Kerr}}/S(0)-1)$ can be of either sign. For the pulsed excitation of 9 ns temporal width, the effect can be predominantly attributed to electronic and thermal contributions^40–42^ and only acts during the presence of the excitation pulse in the medium, as observed in the PIL transients. Usually, the thermal contribution to $n_2$ is negative while the electronic part is positive for liquids^43^. The observed nonlinear index of refraction is an effective value $n_{2,\textrm{eff}}$ combining both effects, which is positive for ethanol, ethylene glycol, and THF, and negative for chloroform, DMF, and DCM.

The steady-state signal at around 400 ns shows the contribution of the thermal lens effect on the sample. As this effect linearly depends on the optical absorption coefficient, chloroform, which has the smallest optical absorption coefficient measured for these liquids at 532 nm, also shows the smallest negative offset. The main contribution to the normalized PIL signal is the pressure wave propagating radially from the excitation volume within the samples, both caused by the optical force (electrostriction) and the heat-deposition-induced pressure gradient.

The analysis presented in Fig. 3 shows that the normalized PIL signal arising from the cuvette walls can be efficiently temporally decoupled from the signal of the liquid. Due to the much larger propagating velocity of sound in the glass (5800 m/s) compared to the one in the liquids (984 m/s–1660 m/s) and due to two-orders of magnitude smaller piezo-optic coefficient, the normalized PIL signal after 100 ns reflects only the response of the liquid.

If one were to design a similar experiment to study solely electrostriction at optical frequencies in low-loss liquids, the following design rules should be taken into account. Geometry: increase the $L/L_{\textrm{g}}$ ratio. Liquid: use liquids which have large $\partial n/\partial p$ and *n*, but small $\partial n/\partial T$ and $A_{\textrm{e}}$. This should be achieved both by choosing the optimum excitation and probe laser wavelength and by setting the temperature of the liquid so as to obtain favorable $\partial n/\partial p$ and $\partial n/\partial T$, which are often temperature and wavelength dependent^44^. Of all six liquids studied in this work, chloroform has the most suitable properties, surpassing those of water^26^.

The measurements presented in Fig. 4a,b (open symbols) are sufficiently decoupled in time that it is possible to retrieve all the free parameters of the theoretical curve. This way low-loss liquids can be characterized by fitting the theory to the experiments. An abundance of unknown properties of the liquid can be obtained with such a pump-probe optical setup.

Equation (3) was first introduced in Ref. ^25^ and later discussed in detail in Ref. ^28^, where it was obtained by using classical dipolar point sources and the Lorentz force density. Here, we have re-obtained the same optical force density by employing the Lagrangian formalism, which provides further theoretical evidence for the validity of the model. Besides, the role of the moving dipoles is much more easily interpreted as the so-called hidden momentum contribution^45,46^ arises naturally. One distinctive feature observed in Ref. ^25^ was the absence of local-field corrections in the form of the Lorentz-Lorenz relation^47^ to the results of electrostriction force density as given by Eq. (3). A phenomenological argumentation has been presented subsequently^27^ to address this observation, where this local-field correction is argued to not occur in the optical regime due to electrostriction causing energetically non-conservative changes in the induced dipole moments through the variation of the material’s local mass density. This behavior is maintained in the current work, i.e., the results seen in Fig. 4 are calculated solely in terms of macroscopic fields. Therefore, the study reported here provides strong evidence that our proposed model for optical force effectively captures the system’s behavior. In particular, applications in liquids where electrostriction is an important effect should benefit from our findings. Such applications can be found, for instance, in laser-induced thermal acoustics^48^ and optical generation of tunable ultrasonic waves^49^. Additionally, electrostriction is known to be related to fluid stability^50,51^. The expression employed for the bulk forces within the liquids, derived from the microscopic Ampère force density, is assumed to represent the total optical force density acting within the samples.

In summary, we have shown that laser-induced optical electrostriction effects can be observed in transparent dielectric liquids, and our experimental observations and their theoretical analyses indicate a remarkable agreement. The results thus pave the way toward a comprehensive definition of the optical force density inside dielectric materials—a crucial concept for modern opto-mechanical applications—and unquestionably contribute to the ongoing resolution of the century-old Abraham-Minkowski controversy.

## Methods

### Derivation of the optical force

The definite knowledge of the form of the optical force is still under debate within the specialized community. Here, we provide for clarity a detailed description of the optical force adopted.

The dielectric medium is described as a continuum of point molecules endowed with electric and magnetic dipoles. The interaction of each molecule with the external fields is given in the laboratory inertial reference frame by the energy *U* as^52^

11 $$\begin{aligned} U = -\textbf{p}(\textbf{E}) \cdot \textbf{E} - \textbf{m}(\textbf{B})\cdot \textbf{B}, \end{aligned}$$

where $\textbf{p}$ and $\textbf{m}$ are the electric and magnetic dipole moments, respectively, which can be induced by the external fields—the electric field $\textbf{E}$ and the magnetic induction field $\textbf{B}$. Notice that the rest mass term of the molecule was not included in Eq. (11). It would ultimately lead to a mass density wave propagating along with the light pulse within the material due to the optical force, according to the mass-polariton quasi-particle theory^4^. Although fundamentally crucial to the Abraham-Minkowski problem, this contribution is very small and hence can be neglected in our current model.

Initially, molecules are assumed to be at rest in the absence of external fields, having electric dipole moment $\textbf{p}_0$ and magnetic dipole moment $\textbf{m}_0$, both given in the rest frame. When the external fields are applied, the interaction will produce optical forces acting on the medium elements which will cause them to become accelerated. Therefore, let us consider these elements acquire the instantaneous and non-relativistic velocity $\textbf{v}$ as measured in the laboratory frame. In such frame, to first order the dipole moments are given as^53^ $\textbf{p} = \textbf{p}_0 + \textbf{v}\times \textbf{m}_0/c^2$ and $\textbf{m} = \textbf{m}_0 - \textbf{v}\times \textbf{p}_0$, where *c* is the speed of light in vacuum. The Lagrangian $\mathcal L$ in the laboratory frame then reads

12 $$\begin{aligned} {\mathcal {L}} = \frac{1}{2}m|\textbf{v}|^2 +\left[ \textbf{p}_0 (\textbf{E})+ \frac{1}{c^2}\textbf{v}\times \textbf{m}_0(\textbf{B})\right] \cdot \textbf{E} +\left[ \textbf{m}_0 (\textbf{B}) - \textbf{v}\times \textbf{p}_0(\textbf{E})\right] \cdot \textbf{B}, \end{aligned}$$

where *m* is the mass of an individual molecule. We consider the induced dipoles to be linear in the corresponding external fields, i.e., we take $\textbf{p}_0 \propto \textbf{E}$ and $\textbf{m}_0 \propto \textbf{B}$, and keep the description for the laboratory frame. This leads to

13 $$\begin{aligned} {\mathcal {L}} = \frac{1}{2} m |\textbf{v}|^2 +\frac{1}{2}\textbf{p}_0 \cdot \textbf{E} + \frac{1}{c^2}(\textbf{v}\times \textbf{m}_0)\cdot \textbf{E} +\frac{1}{2}\textbf{m}_0 \cdot \textbf{B} - (\textbf{v}\times \textbf{p}_0)\cdot \textbf{B}. \end{aligned}$$

From Eq. (13) we are now able to obtain the equations of motion for the dipoles under the external electromagnetic fields. The canonical momentum is ${\varvec{\pi }} = m \textbf{v} - \textbf{p} \times \textbf{B} + \textbf{m} \times \textbf{E}/c^2$, where we have dropped the unnecessary subscript because from now on it is implied we are working exclusively in the laboratory frame. The Euler-Lagrange equations of motion then yield the force $\textbf{F}$ experienced by each medium element as $\textbf{F}= m\,\textrm{d}\textbf{v}/\,\textrm{d}t$, where

14 $$\begin{aligned} m \frac{\,\textrm{d}\textbf{v}}{\,\textrm{d}t}=\frac{1}{2}\varvec{\nabla }(\textbf{p} \cdot \textbf{E} + \textbf{m} \cdot \textbf{B}) + \varvec{\nabla }\left[ \frac{1}{c^2}(\textbf{v}\times \textbf{m})\cdot \textbf{E}-(\textbf{v}\times \textbf{p}) \cdot \textbf{B}\right] +\frac{\,\textrm{d}}{\,\textrm{d}t}\left( \textbf{p}\times \textbf{B}-\frac{1}{c^2}\textbf{m}\times \textbf{E}\right) . \end{aligned}$$

Now, the two terms linear in $\textbf{v}$ in Eq. (14) are neglected as $|\textbf{v}| \ll c$. This yields

15 $$\begin{aligned} \textbf{F}=\frac{1}{2} \varvec{\nabla }(\textbf{p} \cdot \textbf{E} + \textbf{m} \cdot \textbf{B}) +\frac{\,\textrm{d}}{\,\textrm{d}t}\left( \textbf{p}\times \textbf{B}-\frac{1}{c^2}\textbf{m}\times \textbf{E}\right) . \end{aligned}$$

In the point dipole approximation adopted the dipole moments $\textbf{p}$ and $\textbf{m}$ are constant in space. As the dielectric is modeled as a continuum of point molecules, we can transform the microscopic quantities $\textbf{p}$ and $\textbf{m}$ into their macroscopic fields counterparts, the polarization field $\textbf{P}$ and magnetization field $\textbf{M}$ respectively, which is the usual procedure employed in condensed matter systems to smooth the true, microscopic atomic fields. For linear media, these fields are given as $\textbf{P} = \varepsilon _0 (\varepsilon _{\textrm{r}} -1) \textbf{E}$ and $\textbf{M} = ( \mu _{\textrm{r}}-1) \textbf{H}$, where $\textbf{H}$ is the magnetic field, related to the magnetic induction field by $\textbf{H}= (\mu _0\mu _{\textrm{r}})^{-1}\textbf{B}$, $\varepsilon _0$ and $\mu _0$ are the vacuum’s permittivity and permeability, respectively, with the material’s relative analogues denoted by $\varepsilon _{\textrm{r}}$ and $\mu _{\textrm{r}}$. However, We must notice a very important subtlety introduced by this procedure: contrary to $\textbf{p}$ and $\textbf{m}$, the fields $\textbf{P}$ and $\textbf{M}$ present spatial variations. This means that, when switching from the discrete to the continuum description, we must also consider the possibility of spatially varying $\varepsilon _{\textrm{r}}$ and $\mu _{\textrm{r}}$, i.e., the correspondences $\varvec{\nabla } (\textbf{p}\cdot \textbf{E}) \rightarrow V \varvec{\nabla } (\textbf{P}\cdot \textbf{E})$ and $\varvec{\nabla } (\textbf{m}\cdot \textbf{B}) \rightarrow V\varvec{\nabla } (\textbf{M}\cdot \textbf{B})$, where *V* denotes the dielectric’s volume, are not immediately valid. They can work, however, if we simply subtract appropriate terms proportional to the gradient of $\varepsilon _{\textrm{r}}$ and $\mu _{\textrm{r}}$, according to the product rule of derivatives. As $\varvec{\nabla }(\textbf{P}\cdot \textbf{E})=\varepsilon _0(\varepsilon _{\textrm{r}}-1)\varvec{\nabla }|\textbf{E}|^2+\varepsilon _0|\textbf{E}|^2\varvec{\nabla }\varepsilon _{\textrm{r}}$, the correct discrete-to-continuum correspondence for the electric part is then $\varvec{\nabla } (\textbf{p}\cdot \textbf{E}) \rightarrow V [\varvec{\nabla } (\textbf{P}\cdot \textbf{E})-\varepsilon _0 |\textbf{E}|^2\varvec{\nabla }\varepsilon _{\textrm{r}}]$. Similarly, for the magnetic part we have $\varvec{\nabla } (\textbf{m}\cdot \textbf{B}) \rightarrow V[\varvec{\nabla } (\textbf{M}\cdot \textbf{B})-\mu _0|\textbf{H}|^2\varvec{\nabla }\mu _{\textrm{r}}]$. At last, we obtain the so-called microscopic Ampère optical force density^28^, namely

16 $$\begin{aligned} \textbf{f}= & {} -\frac{1}{2}|\textbf{E}|^2\varvec{\nabla }\varepsilon -\frac{1}{2}|\textbf{H}|^2\varvec{\nabla } \mu +\frac{1}{2}\varvec{\nabla }(\textbf{P} \cdot \textbf{E} + \textbf{M} \cdot \textbf{B}) +\frac{n^2-1}{c^2}\frac{\partial }{\partial t}(\textbf{E} \times \textbf{H}). \end{aligned}$$

Here, $\varepsilon = \varepsilon _0 \varepsilon _{\textrm{r}}$, $\mu = \mu _0 \mu _{\textrm{r}}$ and *n* is the refractive index of the medium, given as $n=\sqrt{\varepsilon _{\textrm{r}}\mu _{\textrm{r}}}$. The last term was obtained by substituting the linear relations shown in the last paragraph, recalling that $c = (\varepsilon _0\mu _0)^{-1/2}$. Notice the result in Eq. (16) can also be derived from the Lorentz force density along with the dipolar electromagnetic sources, as shown in detail in Ref. ^28^.

Equation (16) is valid for linear, non-dispersive and lossless dielectrics. The third and fourth terms are the electro- and magnetostriction effects. It is important to point out here that our definition of striction effects is distinct from the one traditionally employed within the photoacoustics community. In the latter, striction forces are described through photo-elastic coefficients as general effects quadratic on the fields—see Refs. ^54–56^ for example. The first and second terms from Eq. (16) are also contained in this approach, as they are related, for example, to stimulated Brillouin scattering within the material. Our definition, on the other hand, includes only bulk contributions because we are working in an optical regime; nevertheless, in homogeneous media the first and second terms from Eq. (16) are important if we are interested in surface deformations as well. For a more detailed discussion on this difference, see Ref. ^26^. At last, the time-derivative term in Eq. (16) is known as Abraham force (density) and is directly related to the optical linear momentum transfer inside the dielectric, lying therefore at the heart of the Abraham-Minkowski problem. It is typically smaller than the striction effects and has not, to our knowledge, been observed at optical frequencies to date.

We consider the incidence of a linearly polarized pulsed Gaussian beam in the fundamental mode onto a low-absorbing sample. In this case, the beam intensity *I*(*r*, *t*) is given by

17 $$\begin{aligned} I(r,t) = Q_0 {\textrm{e}}^{-\frac{2r^2}{w_{{\textrm{e}}} ^2}}h(t), \end{aligned}$$

where $Q_0 = 2 E_{\textrm{ p}}/\pi w_{{\textrm{e}}} ^2$ is the heat source, with $E_{\textrm{ p}}$ and $w_{{\textrm{e}}}$ being the excitation beam energy and waist, respectively. The function *h*(*t*) describes the pulsed time-dependence and is given as $h(t)=t_0^{-1}\textrm{exp}[-(t-\xi )^2/\tau ^2]$, where the time of maximum irradiance and width are denoted by $\xi$ and $\tau$, respectively, and $t_0 = \tau \sqrt{\pi }[1+\textrm{Erf}(\xi /\tau )]/2$ is the normalization parameter given in terms of the error function $\textrm{Erf}(x)$.

Equation (16) allows us to write the optical force source term in Eq. (6). For bulk non-magnetic fluids under optical excitation, only the third term in Eq. (3) contributes significantly, yielding $\textbf{f}= \varepsilon _0(n^2 -1)\varvec{\nabla } |\textbf{E}|^2/2$. In our case, this simplifies to

18 $$\begin{aligned} \textbf{f}(r,t) = -F_0 r{\textrm{e}}^{-\frac{2 r^2}{w_{{\textrm{e}}} ^2}}h(t)\hat{\textbf{r}}, \end{aligned}$$

where $F_0 = 16(n-1)E_{\textrm{ p}}/[c(n+1)\pi w_{{\textrm{e}}} ^4]$ and $\hat{\textbf{r}}$ denotes the radial direction. Notice that this force density points towards the center of the beam, indicating compression of the fluid, as expected for the electrostriction effect. In terms of beam intensity, the magnitude of Eq. (18) is

19 $$\begin{aligned} f(r,t) = \frac{8r}{cw_{{\textrm{e}}} ^2}\frac{n-1}{n+1} I(r,t), \end{aligned}$$

which is the same magnitude of Eq. (7).

### Semi-analytical solutions

With Eq. (18), the coupled equations for temperature and pressure dynamics possess semi-analytical solutions^26^ stemming from integral transform methods. They are separately given as

20 $$\begin{aligned} T(r,t)= \frac{Q_0 A_{\textrm{e}}}{4\rho _{\textrm{m}} c_p}\frac{1}{[1+\textrm{Erf}(\xi /\tau )]} \int _0^{\infty } w_{{\textrm{e}}}^2e^{-\frac{1}{8}w_{{\textrm{e}}}^2 \alpha ^2} \chi (D \alpha ^2)J_0(\alpha r) \alpha d\alpha \end{aligned}$$

and

21 $$\begin{aligned} p(r,t)= \frac{1}{4}\int _0^{\infty } w_{{\textrm{e}}}^2 e^{-\frac{1}{8}w_{{\textrm{e}}}^2\alpha ^2}~g(\alpha ,t)J_0(\alpha r) \alpha d\alpha , \end{aligned}$$

where $D = k/(\rho _{\textrm{m}}c_p)$ is the thermal diffusivity, $J_0$ is the 0-th order Bessel function of the first kind and

22 $$\begin{aligned} g(\alpha ,t)=g_{\textrm{ESW}}(\alpha ,t)+g_{\textrm{TEW}}(\alpha ,t), \end{aligned}$$

where

23 $$\begin{aligned} g_{\textrm{ESW}}(\alpha ,t)=\frac{F_0 w_{{\textrm{e}}}^2 (\mathrm i c_{\textrm{ s}} \alpha ) }{8 [1+\textrm{Erf}(\xi /\tau )]}\left[ \chi (-\mathrm i c_{\textrm{ s}} \alpha )-\chi (\mathrm i c_{\textrm{ s}} \alpha )\right] \end{aligned}$$

and

24 $$\begin{aligned} g_{\textrm{TEW}}(\alpha ,t)=\frac{Q_0 A_{\textrm{e}}}{c_p}\frac{\beta }{\tau }\frac{c_{\textrm{ s}}}{[1+\textrm{Erf}(\xi /\tau )]} \frac{1}{2\alpha } \left( -\frac{4 \sin (c_{\textrm{ s}} t \alpha )}{\sqrt{\pi }}e^{-\xi ^2/\tau ^2} +\frac{}{} c_{\textrm{ s}} \tau \alpha \left[ \chi (\mathrm i c_{\textrm{ s}} \alpha )+\chi (-\mathrm i c_{\textrm{ s}} \alpha )\right] \right) , \end{aligned}$$

with

25 $$\begin{aligned} \chi (x)=\left[ \textrm{Erf}\left( \frac{\xi }{\tau }+\frac{x \tau }{2} \right) -\textrm{Erf}\left( \frac{\xi -t}{\tau }+\frac{x \tau }{2} \right) \right] e^{-\left[ x(t-\xi )-\frac{x^2 \tau ^2}{4}\right] }. \end{aligned}$$

Here we assume that $L \ll A_{{\textrm{e}}} ^{-1}$ (the samples have low optical absorption coefficient) and infinite radial boundary conditions. This last assumption holds because, at the nanosecond time scale, the thermal lens measurement is unaffected since the generated pressure waves only reach the edge of the cuvette after the measurement ends.

### Photo-induced lensing technique

The PIL signal *S*(*t*) given in Eq. (9) is monitored through the time-resolved photo lensing experimental technique depicted in Fig. 1. The excitation beam is generated from a Q-switched Nd:YAG laser at $\lambda _{{\textrm{e}}} = 532$ nm in TEM_00_ mode with parameters $\xi = 15$ ns and $\tau = 9$ ns describing the pulsed time-dependence. This beam is focused onto the low-absorbing liquid sample of $L=5$ mm thickness by using a lens with $f=0.75$ m focal distance. The probe beam, on its turn, is generated from a He-Ne laser operating in CW regime at $\lambda _{\textrm{ p}}=632.8$ nm and consists of a low-irradiance TEM_00_ beam. It is focused by a lens with a $f=0.30$ m focal distance at 5 cm from the sample and travels almost colinearly with the excitation beam. The detection of the intensity of this probe beam, *S*(*t*), is carried out by a pinhole-laser line filter-photodetector assembly 5 m from the sample (the far-field region). The filter line is employed to avoid the undesired detection of ambient light and the excitation beam and the photodetector has 200 MHz bandwidth. A digital oscilloscope records the signal. The excitation beam was used to trigger the oscilloscope by using another photodetector (same as the probe sensor) at a repetition frequency of 10 Hz. An actively damped optical table was used to align the setup configuration. The temperature of the samples was kept at $298.15\pm 0.05$ K by a heating/cooling unit and a temperature controller. The excitation and probe beam radii are $w_{{\textrm{e}}} = 116$ $\mu$m and $w_{\textrm{ p}} = 269$ $\mu$m, respectively, and were measured with a beam profile camera, while the laser energy $E_{\textrm{ p}}$ was measured using a pyroelectric energy sensor (shown in Fig. 4). The experimental parameter $V= 6.5$. The statistical uncertainties are smaller than 1% and correspond to the standard deviation of the mean throughout the experiments. At least 15 measurements were performed for each sample, with averaging performed over 512 transients to reduce noise.

### Supplementary Information

Supplementary Information.

## Data Availability

The datasets generated during the current study are available from the corresponding author upon reasonable request.
