# Supplementary material for: Towards a comprehensive characterization of spatio-temporal dependence of light-induced electromagnetic forces in dielectric liquids
Source: Sci Rep. 2024 Mar 7;14:5595. doi: 10.1038/s41598-024-56176-1 (PMC10920765; doi:10.1038/s41598-024-56176-1)
Supplement: Supplementary file 1 — Supplementary Information. [file 41598_2024_56176_MOESM1_ESM.pdf]

Supplementary information for "Towards a comprehensive characterization of spatio-temporal dependence of light-induced electromagnetic forces in dielectric liquids"

N. G. C. Astrath, E. V. Bergmann, B. Anghinoni, G. A. S. Flizikowski, A. Novatski, C. Jacinto, T. Požar, M. Kalin, L. C. Malacarne and M. L. Baesso.

Physical properties of the liquids

The physical properties of the liquids studied are shown in Table S1.

Table S1. Acoustic, thermal and optical properties of the liquids at 25°C.

Parameter	n	c_s	$-\partial n/\partial T$	$\partial n/\partial p$	A_e	$n_{2,\mathrm{eff}}$
Units		m/s	10^{-4}/K	10^{-10}/Pa	1/m	$10^{-20}\mathrm{m}^2/\mathrm{W}$
Source	Ref. [1]	Ref. [1]	Ref. [1]	Ref. [1]	Measured	Measured
Ethanol	1.35962	1144	4.41	3.95	0.0392	1.36
Chloroform	1.44222	984	6.70	3.71	0.0026	-1.79
Ethylene glycol	1.42782	1660	3.06	1.79	0.0290	1.82
Dimethylformamide (DMF)	1.42782	1457	4.93	2.61	0.0196	-6.47
Dichloromethane (DCM)	1.42024	1071	6.92	3.12	0.0046	-3.72
Tetrahydrofuran (THF)	1.40414	1284	5.73	3.16	0.0095	1.60
Fused silica	1.5	5800	0.096	0.031	0.0050	1.70

Small-signal approximation

Small-signal approximation of the PIL signal $S(t)$ was assumed to simplify Eq. (9) to Eq. (10). This linearization is justified because we have numerically tested the validity of the approximation—namely, we have separately calculated each term within brackets on the right-hand side of this approximate relation

$$\left(\frac{S(t)}{S(0)}-1\right) \approx \left(\frac{S_{\mathrm{TD}}(t)}{S(0)}-1\right)+\left(\frac{S_{\mathrm{ESW}}(t)}{S(0)}-1\right)+\left(\frac{S_{\mathrm{TEW}}(t)}{S(0)}-1\right)+\left(\frac{S_{\mathrm{Kerr}}(t)}{S(0)}-1\right)$$

and have shown that the sum of all four terms on the right-hand side very closely matches the total normalized PIL signal as given in the bracket on the left-hand side. Here

$$S(t)=\left|\int_0^\infty \frac{2r}{w_{\mathrm{p}}^2}\exp\left[-(1+\mathrm{i}V)\frac{r^2}{w_{\mathrm{p}}^2}-\mathrm{i}\Phi(r,t)\right]\mathrm{d}r\right|^2,$$

is the total, time-dependent PIL signal as given in Eq. (9),

$$S(0)=\left|\int_0^\infty \frac{2r}{w_{\mathrm{p}}^2}\exp\left[-(1+\mathrm{i}V)\frac{r^2}{w_{\mathrm{p}}^2}\right]\mathrm{d}r\right|^2$$

is the initial PIL signal before the excitation,

$$S_{\mathrm{TD}}(t)=\left|\int_0^\infty \frac{2r}{w_{\mathrm{p}}^2}\exp\left[-(1+\mathrm{i}V)\frac{r^2}{w_{\mathrm{p}}^2}\right]\exp\left[-\mathrm{i}\frac{2\pi}{\lambda_{\mathrm{p}}}L\frac{\partial n}{\partial T}T(r,t)\right]\mathrm{d}r\right|^2$$

is the PIL signal solely caused by the thermal deposition,

$$S_{\mathrm{ESW}}(t)=\left|\int_0^\infty \frac{2r}{w_{\mathrm{p}}^2}\exp\left[-(1+\mathrm{i}V)\frac{r^2}{w_{\mathrm{p}}^2}\right]\exp\left[-\mathrm{i}\frac{2\pi}{\lambda_{\mathrm{p}}}L\frac{\partial n}{\partial p}p_{\mathrm{ESW}}(r,t)\right]\mathrm{d}r\right|^2$$

is the piezo-optic PIL signal solely caused by the propagation of the electrostriction-generated elastic waves (ESW),

$$S_{\mathrm{TEW}}(t)=\left|\int_0^\infty \frac{2r}{w_\mathrm{p}^2}\exp\left[-(1+\mathrm{i}V)\frac{r^2}{w_\mathrm{p}^2}\right]\exp\left[-\mathrm{i}\frac{2\pi}{\lambda_\mathrm{p}}L\frac{\partial n}{\partial p}p_{\mathrm{TEW}}(r,t)\right]\mathrm{d}r\right|^2$$

is the piezo-optic PIL signal solely caused by the launch of the thermally-generated elastic waves (TEW), and lastly

$$S_{\mathrm{Kerr}}(t)=\left|\int_0^\infty \frac{2r}{w_\mathrm{p}^2}\exp\left[-(1+\mathrm{i}V)\frac{r^2}{w_\mathrm{p}^2}\right]\exp\left[-\mathrm{i}\frac{2\pi}{\lambda_\mathrm{p}}L\frac{\partial n}{\partial I}I(r,t)\right]\mathrm{d}r\right|^2$$

is the short-lived PIL signal solely due to Kerr effect.

One example of such a numerical test is given in Fig. S1.

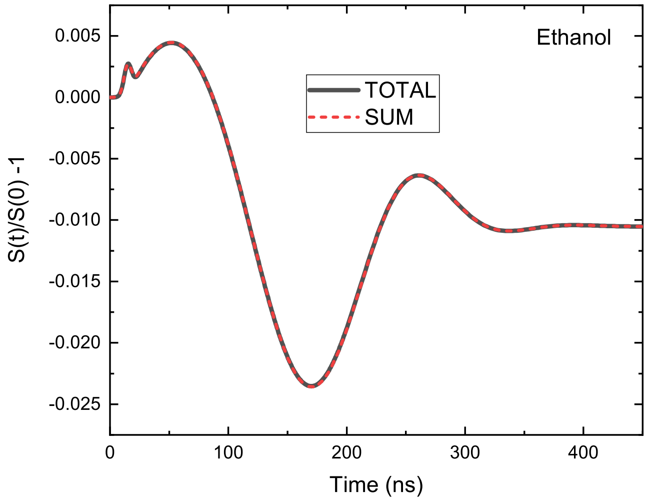

Figure S1. Simulation of the time-dependent PIL transients for ethanol. The transients show the total intensity signal calculated using the full description of the normalized PIL signal (TOTAL) and the SUM of all the individual contributions from each effect separately.

References

1. Bergmann, E. V. *et al.* Optoacoustic detection of nanosecond time scale photoinduced lensing effects in liquids. *J. Appl. Phys* **134**, 165103, DOI: 10.1063/5.0172822 (2023).
